# Leaf Lateral Asymmetry in Morphological and Physiological Traits of Rice Plant

**DOI:** 10.1371/journal.pone.0129832

**Published:** 2015-06-08

**Authors:** Shen Yuan, Yong Li, Shaobing Peng

**Affiliations:** National Key Laboratory of Crop Genetic Improvement, Ministry of Agriculture Key Laboratory of Crop Ecophysiology and Farming System in the Middle Reaches of the Yangtze River, College of Plant Science and Technology, Huazhong Agricultural University, Wuhan, Hubei, China; National Taiwan University, TAIWAN

## Abstract

Leaf lateral asymmetry in width and thickness has been reported previously in rice. However, the differences between the wide and narrow sides of leaf blade in other leaf morphological and physiological traits were not known. This study was conducted to quantify leaf lateral asymmetry in leaf width, leaf thickness, specific leaf weight (SLW), leaf nitrogen (N) concentration based on dry weight (Nw) and leaf area (Na), and chlorophyll meter reading (SPAD). Leaf morphological and physiological traits of the two lateral halves of the top three leaves at heading stage were measured on 23 rice varieties grown in three growing seasons in two locations. Leaf lateral asymmetry was observed in leaf width, leaf thickness, Nw, Na, and SPAD, but not in SLW. On average, the leaf width of the wide side was about 17% higher than that of the narrow side. The wide side had higher leaf thickness than the narrow side whereas the narrow side had higher Nw, Na, and SPAD than the wide side. We conclude that the narrow side of leaf blade maintained higher leaf N status than the wide side based on all N-related parameters, which implies a possibility of leaf lateral asymmetry in photosynthetic rate in rice plant.

## Introduction

Rice (*Oryza sativa* L.) is one of the most important staple foods in the world, providing 35–60% of the dietary calories consumed by more than 3 billion people [1]. Increasing rice yields on existing land is a primary strategy for increasing rice production in order to meet the demand of growing population [2]. Rice leaves have many functions such as light interception, photosynthesis, and assimilate storage; therefore, leaf morphological and physiological traits have large effects on rice grain yield [3].

The optimum leaf length, width, thickness, angle, and area were considered in developing high-yielding rice varieties through ideotype breeding [4]. The varieties with high yield potential usually have leaves that were erect, short, narrow, thick, and dark green [5]. A thick leaf usually has higher chlorophyll, nitrogen (N), and photosynthetic enzymes content per unit leaf area [6,7]. Therefore, there is a positive correlation between single-leaf net photosynthetic rate and leaf thickness in rice [8]. Furthermore, a thick leaf has less tendency to expand horizontally and a greater tendency to be erect [9]. An accurate measurement of leaf thickness is difficult, therefore, specific leaf weight (SLW) defined as the ratio of dry weight to area of leaf blade is used as an indicator of leaf thickness [10].

Single-leaf net photosynthetic rate and leaf N concentration are highly correlated in rice [11], therefore, maintaining adequate leaf N throughout growing season is crucial for achieving high crop yields [12]. Leaf N status has been used to guide the timing and amount of fertilizer-N application to improve rice yield and N use efficiency. Leaf N concentration can be expressed based on dry weight (Nw) or leaf area (Na). Because of a close relationship between leaf N and chlorophyll concentrations in rice leaves, a chlorophyll meter (SPAD) is used for estimating leaf N concentration [13].

Rice leaf blade is divided by midrib into two lateral halves. Ishii [14] reported that the width of each lateral half in rice leaves (i.e. distance from the midrib to the leaf edge) was not equal: one side was wider and the other narrower. *Leaf lateral symmetry 1* (*LSY1*) gene was reported to cause rice leaf lateral asymmetry by deleting a part or whole of one lateral half or forming additional midrib in one lateral half [15]. Leaf lateral asymmetry in width has been reported in many species in addition to rice such as wheat [16], oak [17], Chinese evergreen [18], and birch [19]. In these studies, leaf lateral asymmetry in width was used to quantify fluctuating asymmetry in order to study the effects of environmental and genetic factors on developmental stability.

Chen et al. [20] reported differences in leaf thickness between the two sides of leaf blade in rice. However, leaf lateral asymmetry in SLW and leaf N status was not reported in rice. In this study, we measured leaf width, thickness, SLW, leaf N concentration, and SPAD of the two lateral halves of the top three leaves at heading stage on 23 rice varieties grown in three growing seasons in two locations. The objectives were to (1) quantify the differences between the wide and narrow sides of leaf blade in the morphological and physiological traits and (2) compare the magnitudes of leaf lateral asymmetry across leaf traits and leaf positions.

## Materials and Methods

### Field experiments

Four field experiments were conducted in 2014 at Xiazhouyu Village, Dajin Township, Wuxue County (29°51′N, 115°33′E, 51 m altitude) and the experimental farm of Huazhong Agricultural University, Wuhan City (30°35′N, 114°17′E, 23.3 m altitude), Hubei Province, China. The field in Wuxue is owned by Caijin Xu, a farmer from Xiazhouyu Village. The field in Wuhan is owned by Huazhong Agricultural University. No specific permissions were required for conducting these field experiments because they are rice fields and our field studies did not involve endangered or protected species. For the Wuxue site, three field experiments were conducted in the early (April to July), middle (May to October), and late (July to November) growing seasons. Only one field experiment was conducted during the middle growing season in Wuhan. Four and 8 varieties were used in the early and late growing seasons, respectively, in Wuxue (Table 1). The experiments in the middle growing season had 14 varieties in both Wuxue and Wuhan. Varieties were arranged in a complete randomized block design with four replications in all four experiments. In the early growing season, 28-d-old seedlings were transplanted on 3 May, whereas 20-d-old seedlings were transplanted on 29 July in the late growing season. Hill spacing was 0.20 × 0.20 m in both early and late growing seasons. Twenty five-d-old seedlings were transplanted at the hill spacing of 0.17 × 0.27 m on 17 June for the middle growing season in both locations. In all four experiments, transplanting was done with 2 seedlings per hill and plot size was 30 m^2^.

**Table 1:** General information on the rice varieties grown in the early (April to July), middle (May to October), and late (July to November) growing seasons in Wuxue County and Wuhan City, Hubei Province, China in 2014.

Variety	Location	Season	Subspecies	Type
Liangyou287	Wuxue	Early/Late	Indica	Hybrid
Ezao17	Wuxue	Early/Late	Indica	Inbred
Ezao18	Wuxue	Early/Late	Indica	Inbred
Zhongjiazao17	Wuxue	Early/Late	Indica	Inbred
Huanghuazhan	Wuxue/Wuhan	Middle	Indica	Inbred
Yangliangyou6	Wuxue/Wuhan	Middle	Indica	Hybrid
Zhuliangyou35	Wuxue/Wuhan	Middle	Indica	Hybrid
Zhongzu14	Wuxue/Wuhan	Middle	Indica	Hybrid
5A×R49	Wuxue/Wuhan	Middle	Indica	Hybrid
9you6	Wuxue/Wuhan	Middle	Indica	Hybrid
Guangliangyou5	Wuxue/Wuhan	Middle	Indica	Hybrid
Hanyou73	Wuxue/Wuhan	Middle	Indica	Hybrid
Huiliangyou630	Wuxue/Wuhan	Middle	Indica	Hybrid
Quanyou982	Wuxue/Wuhan	Middle	Indica	Hybrid
Jinkeyou651	Wuxue/Wuhan	Middle	Indica	Hybrid
Rongfengyou41	Wuxue/Wuhan	Middle	Indica	Hybrid
Wuyouhang1573	Wuxue/Wuhan	Middle	Indica	Hybrid
Yungeng29	Wuxue	Middle	Japonica	Hybrid
Huiliangyou858	Wuhan	Middle	Indica	Hybrid
Xiangzao45	Wuxue	Late	Indica	Inbred
Xiangzaoxian32	Wuxue	Late	Indica	Inbred
Wandao143	Wuxue	Late	Indica	Inbred
Xiangzaoxian6	Wuxue	Late	Indica	Inbred

To avoid nutrient deficiency during the entire growing season, sufficient amounts of N, phosphorus (P), and potassium (K) were applied in all four experiments. Nitrogen in the form of urea was applied at basal, tillering, and panicle initiation with the total N rate of 176 kg ha^-1^ in the early growing season, 154 kg ha^-1^ in the late growing season, and 100 kg ha^-1^ in the middle growing season in both locations. Phosphorus in the form of calcium superphosphate was applied only at basal with the P rate of 31 kg ha^-1^ in the early growing season, 24 kg ha^-1^ in the late growing season, and 40 kg ha^-1^ in the middle growing season in both locations. Potassium in the form of potassium chloride was applied at basal and panicle initiation with the total K rate of 93 kg ha^-1^ in the early growing season, 97 kg ha^-1^ in the late growing season, and 100 kg ha^-1^ in the middle growing season in both locations. Basal application of fertilizers was manually broadcast and incorporated 1 d before transplanting. Weeds, diseases, and insects were intensively controlled throughout the entire growing season according to farmers’ practices.

### Sampling and measurements

Three representative plants were selected at heading stage from each replication for the measurements of leaf traits. For each plant, measurements were done on the top three leaves (i.e. flag, -2^nd^, and -3^rd^ leaves). SPAD readings were taken in situ on the top three leaves of selected plants. On each side of midrib, 7 SPAD readings were taken at 1/8, 1/4, 3/8, 1/2, 5/8, 3/4, and 7/8 positions along leaf length using a chlorophyll meter (SPAD-502, Soil-Plant Analysis Development, Konika Minolta, Japan). The 7 SPAD readings were averaged to obtain SPAD values for each side of leaf blade.

After the measurement of SPAD, the three plants with roots were sampled and placed in a plastic bag containing small amount of water for preventing leaf rolling. The sampled plants were transferred immediately to the laboratory where leaf width and thickness were measured. The top three leaves were detached and the leaf width and thickness were measured on the same positions where SPAD reading was taken. The measurement of leaf width on each side of midrib was taken from leaf edge to midrib using the Digital Calipers (YT-7201, YATO Co., Ltd, Germany). Wide and narrow sides of the leaf blade were designated based on their width. Leaf thickness was measured with a displacement sensor (YI-20030A, China Jiliang University) as described by Li et al. [21]. The pressure exerted by this sensor to leaf blade surface was only 0.1 Newton to reduce the variability in leaf thickness measurement [21]. Because leaf thickness tends to increase near midrib, the probe for leaf thickness measurement was placed in the middle point between leaf edge and midrib. In addition, the tip of the probe was spherical in order to avoid large veins during the measurement of leaf thickness. The air temperature in the laboratory was maintained at 26°C to reduce the effect of temperature on leaf thickness [22]. The 7 measurements of leaf width and thickness were averaged to obtain mean values for each side of leaf blade.

Then, each leaf was sliced along the leaf length to remove midrib from all top three leaves manually. For each leaf position, the wide and narrow sides of leaf blade of the three plants were pooled separately to measure leaf area, dry weight, and N concentration of each lateral half. Leaf area was measured by a leaf area meter (LI-3200, Li-Cor, Lincoln, NE). Dry weight was determined after oven-drying at 80°C to constant weight. SLW was calculated as the ratio of dry weight to leaf area. Nw was determined by an Elemental analyzer (Elementar vario MAX CNS/CN, Elementar Trading Co., Ltd, Germany). The Na was calculated by the following equation:

$$Na=\frac{Nw\times SLW}{100}$$

### Data analysis

Statistical analysis was done separately for each leaf position. For leaf SPAD, width, and thickness, the values of the three plants were averaged to represent each replication. Analysis of variance was done for all parameters. Significance of differences between the wide and narrow sides in these parameter were tested by least significant difference at P = 0.05 (LSD_0.05_) for each experiment.

## Results

**Fig 1:**
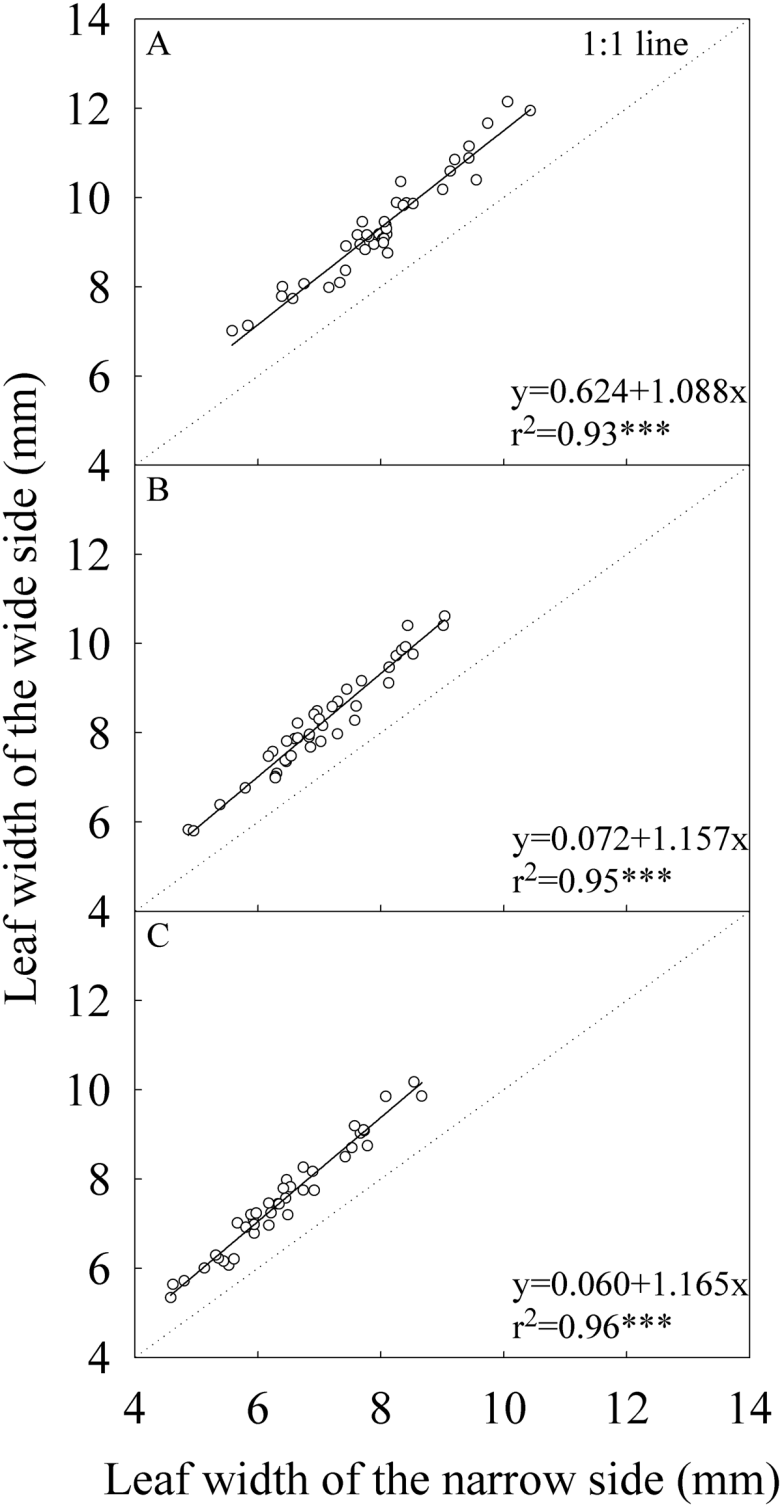
Correlation between the wide and narrow sides of leaf blade in leaf width. (A) The flag, (B) The -2^nd^, and (C) The -3^rd^ leaves at heading stage. Each data point represents the mean of the three plants and four replications. Data (n = 40) were from the four field experiments conducted in three growing seasons in two locations in 2014.

There was statistically significant difference in width between the two lateral halves of leaf blade for all top three leaves across growing seasons and locations (Table 2). The wide side was 16.6%, 16.8%, and 17.4% wider than the narrow side for the flag, -2^nd^, and -3^rd^ leaves, respectively. The absolute difference in leaf width between the wide and narrow sides was increasing as the leaf width increased (Fig 1).

**Table 2:** The leaf width, thickness, and specific leaf weight (SLW) of the wide and narrow sides of leaf blade in the top three leaves at heading stage.

Location	Season	Flag		-2^nd^		-3^rd^		Average	
		Wide	Narrow	Wide	Narrow	Wide	Narrow	Wide	Narrow
					Width (mm)				
Wuxue	Early	8.83 a	7.53 b	7.40 a	6.33 b	6.54 a	5.56 b	7.59 a	6.48 b
Wuxue	Middle	8.97 a	7.75 b	7.88 a	6.81 b	7.22 a	6.20 b	8.02 a	6.92 b
Wuhan	Middle	10.10 a	8.64 b	8.95 a	7.65 b	8.35 a	7.10 b	9.13 a	7.79 b
Wuxue	Late	9.03 a	7.74 b	8.00 a	6.76 b	7.27 a	6.12 b	8.10 a	6.87 b
	Mean	9.36 A	8.03 B	8.22 A	7.04 B	7.55 A	6.43 B	8.38 A	7.17 B
					Thickness (μm)				
Wuxue	Early	166.1 a	134.5 b	158.5 a	130.6 b	157.3 a	128.9 b	160.6 a	131.3 b
Wuxue	Middle	199.0 a	159.2 b	178.4 a	144.5 b	171.5 a	137.0 b	183.0 a	146.9 b
Wuhan	Middle	180.2 a	146.3 b	171.3 a	134.8 b	164.2 a	128.4 b	171.9 a	136.5 b
Wuxue	Late	135.7 a	110.0 b	124.7 a	103.1 b	120.0 a	98.3 b	126.8 a	103.8 b
	Mean	176.6 A	142.4 B	163.2 A	131.5 B	157.6 A	125.7 B	165.8 A	133.2 B
					SLW (g m^-2^)				
Wuxue	Early	44.8 a	42.2 b	41.6 a	40.6 a	39.3 a	38.5 a	41.9 a	40.5 b
Wuxue	Middle	54.4 a	53.8 a	46.2 a	46.2 a	42.2 a	42.7 a	47.6 a	47.6 a
Wuhan	Middle	49.6 a	47.2 b	43.0 a	41.7 a	39.5 a	38.5 a	44.0 a	42.5 b
Wuxue	Late	42.3 a	41.1 a	36.1 a	36.3 a	32.8 a	34.5 a	37.1 a	37.3 a
	Mean	49.3 A	47.8 B	42.6 A	42.1 A	39.1 A	39.2 A	43.7 A	43.1 A

The experiments were conducted in the early, middle, and late growing seasons in Wuxue County and Wuhan City, Hubei Province, China in 2014.

Within a row for each leaf position, means followed by different letters are significantly different according to the least significant difference (LSD) at the 0.05 probability level.

**Fig 2:**
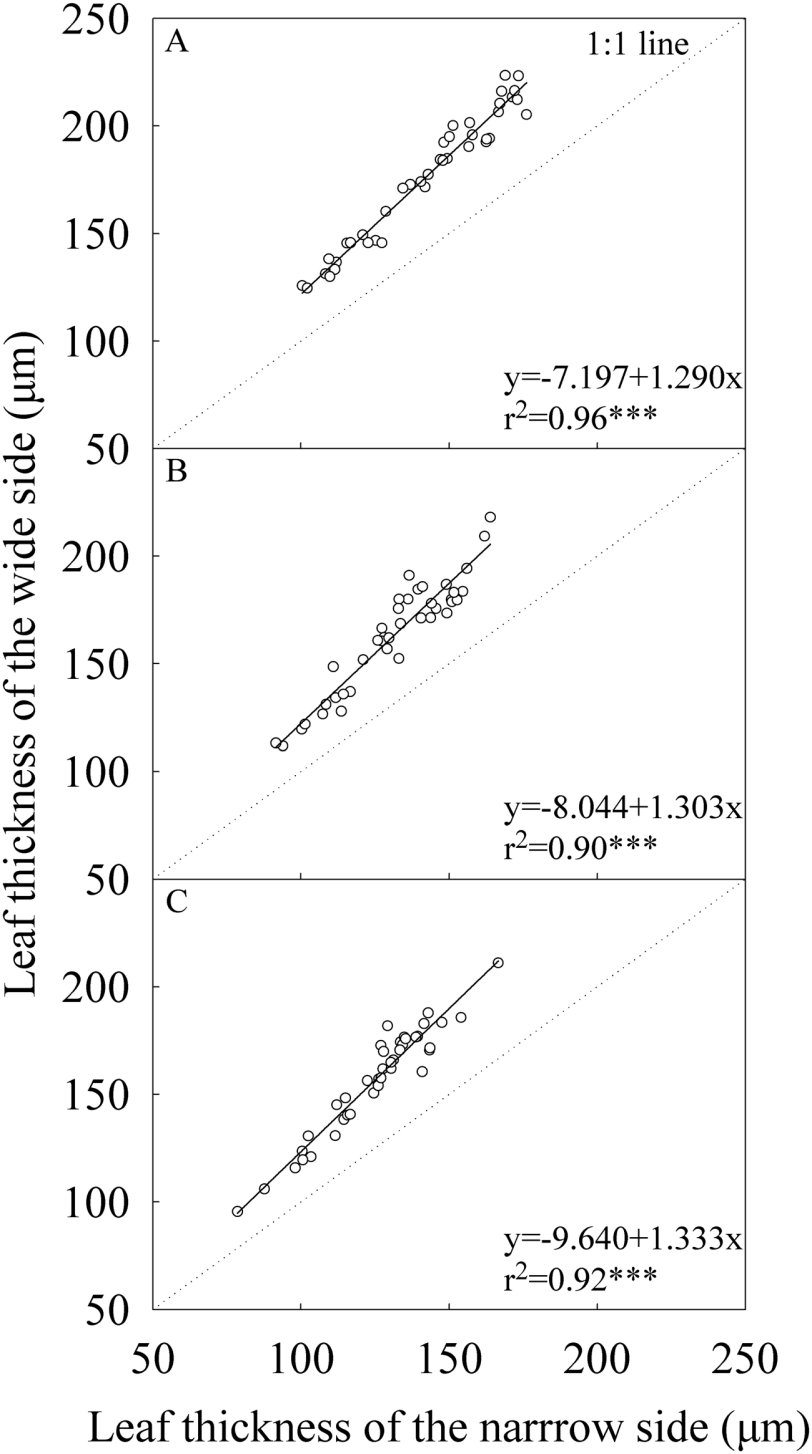
Correlation between the wide and narrow sides of leaf blade in leaf thickness. (A) The flag, (B) The -2^nd^, and (C) The -3^rd^ leaves at heading stage. Each data point represents the mean of the three plants and four replications. Data (n = 40) were from the four field experiments conducted in three growing seasons in two locations in 2014.

The wide and narrow sides had statistically significant difference in leaf thickness for all top three leaves across growing seasons and locations (Table 2). Leaf thickness ranged from 96.4 to 223.5 μm for the wide side and from 78.4 to 168.9 μm for the narrow side across the four experiments (Fig 2). On average, the leaf thickness of the wide side was 24.0%, 24.1%, and 25.4% higher than that of the narrow side for the flag, -2^nd^, and -3^rd^ leaves, respectively (Table 2). The absolute and relative difference in leaf thickness between the wide and narrow sides was increasing as the leaf thickness increased (Fig 2).

**Fig 3:**
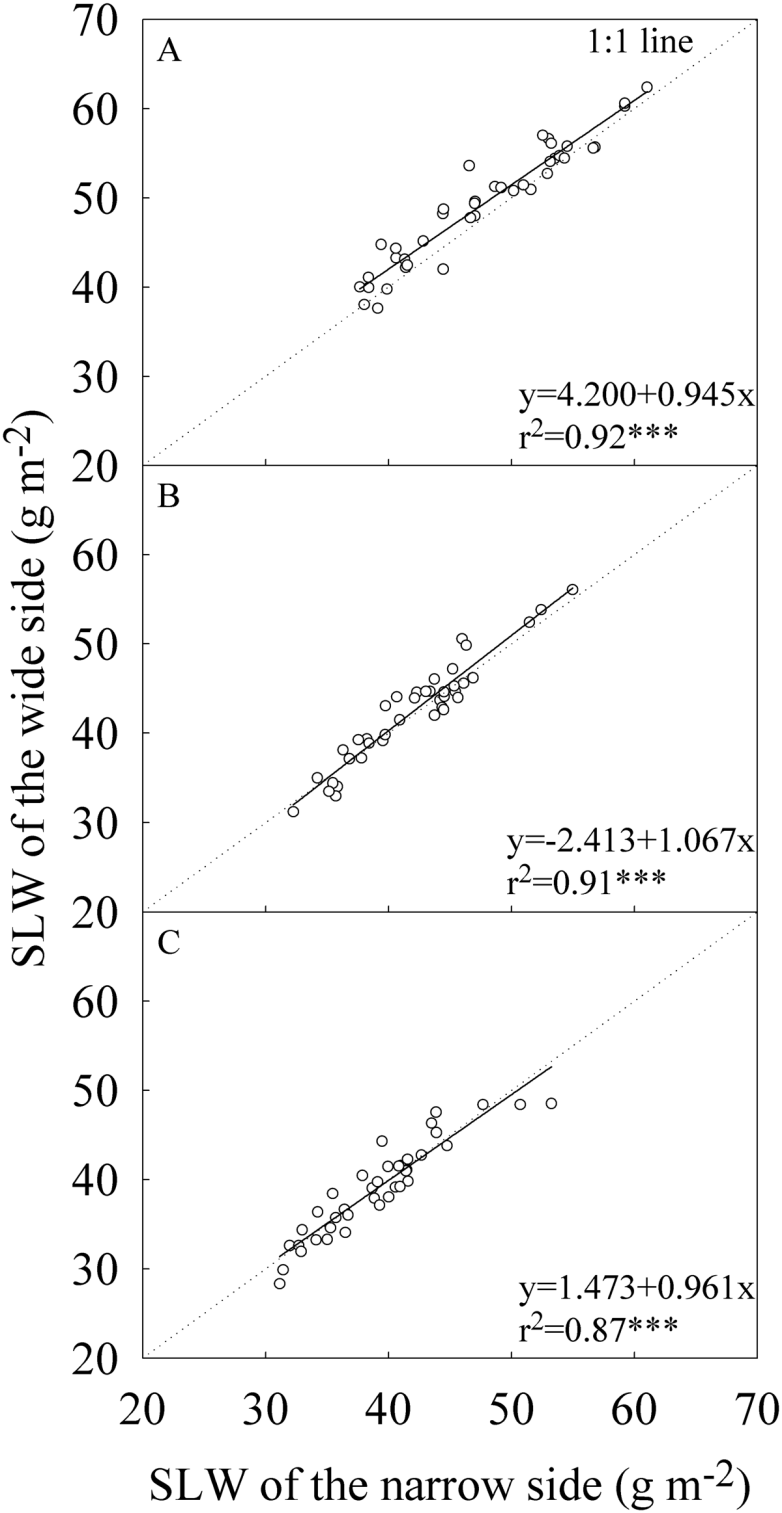
Correlation between the wide and narrow sides of leaf blade in specific leaf weight (SLW). (A) The flag, (B) The -2^nd^, and (C) The -3^rd^ leaves at heading stage. Each data point represents the mean of the four replications. Data (n = 40) were from the four field experiments conducted in three growing seasons in two locations in 2014.

There was no significant difference between the wide and narrow sides in SLW for the -2^nd^ and -3^rd^ leaves in all four experiments (Table 2 and Fig 3). For the flag leaf, the wide side had statistically and significantly higher SLW than the narrow side only for the early growing season in Wuxue and middle growing season in Wuhan (Table 2). Averaged across the four experiments, SLW of the wide side was only 3% higher than that of the narrow side in the flag leaf.

Overall, flag leaf had the highest leaf width, thickness, and SLW followed by -2^nd^ and -3^rd^ leaves (Table 2). All top three leaves in the middle growing season had the highest leaf thickness and SLW followed by the early and late growing seasons. No significant difference was observed in leaf width among the three growing seasons.

**Fig 4:**
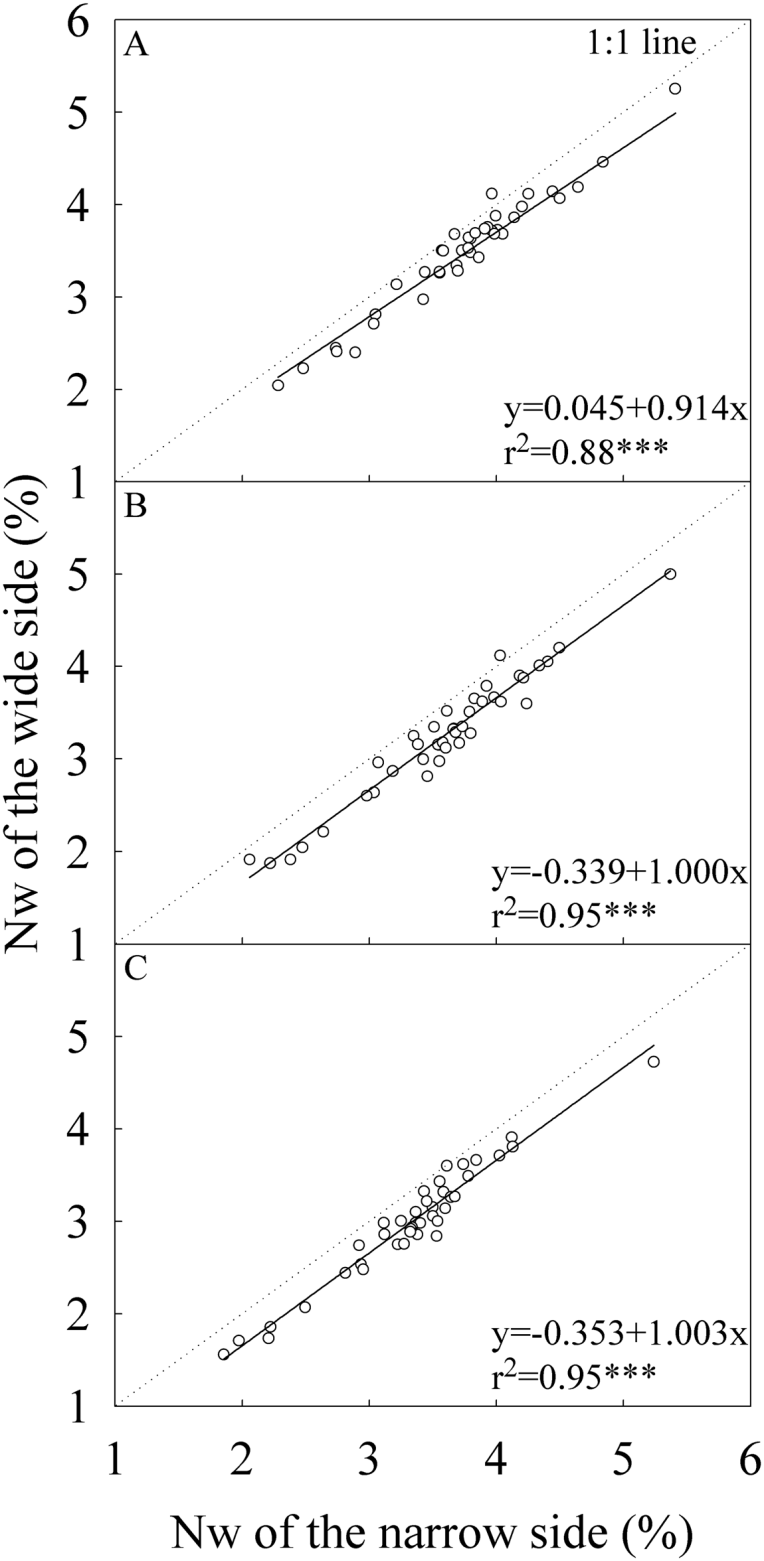
Correlation between the wide and narrow sides in dry weight-based nitrogen concentration (Nw). (A) The flag, (B) The -2^nd^, and (C) The -3^rd^ leaves at heading stage. Each data point represents the mean of the four replications. Data (n = 40) were from the four field experiments conducted in three growing seasons in two locations in 2014.

**Fig 5:**
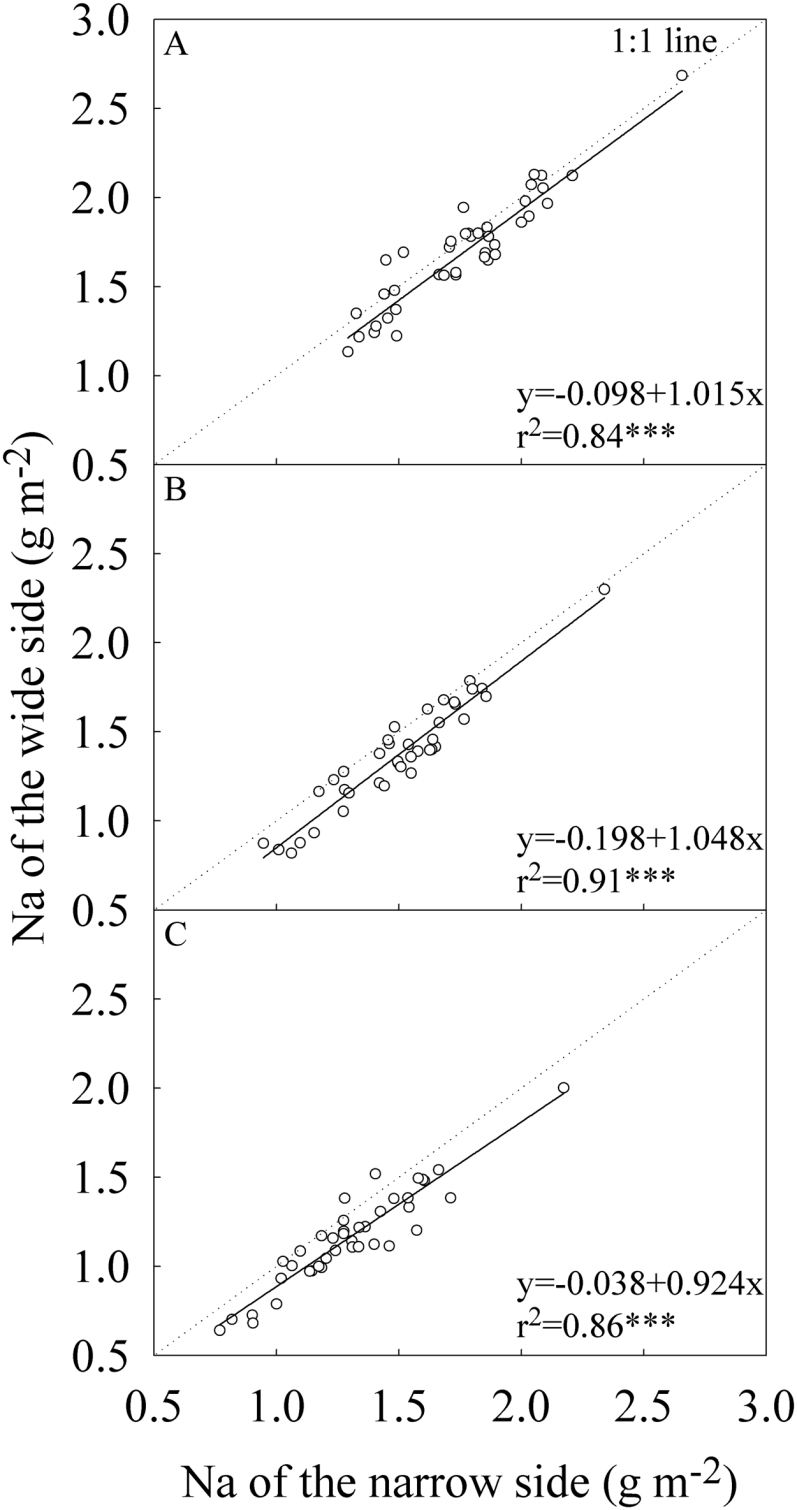
Correlation between the wide and narrow sides in leaf area-based nitrogen concentration (Na). (A) The flag, (B) The -2^nd^, and (C) The -3^rd^ leaves at heading stage. Each data point represents the mean of the four replications. Data (n = 40) were from the four field experiments conducted in three growing seasons in two locations in 2014.

**Fig 6:**
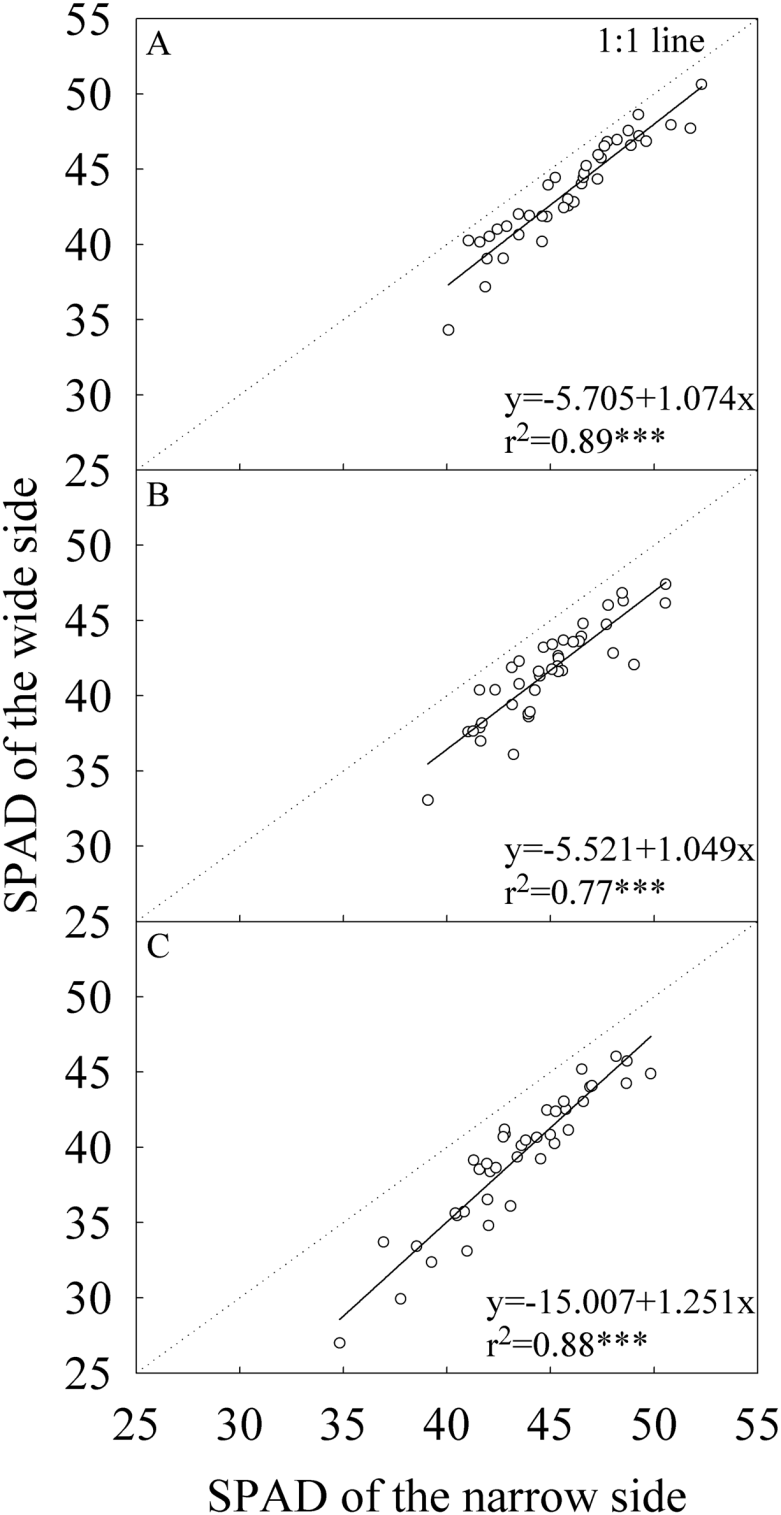
Correlation between the wide and narrow sides of leaf blade in chlorophyll meter reading (SPAD). (A) The flag, (B) The -2^nd^, and (C) The -3^rd^ leaves at heading stage. Each data point represents the mean of the three plants and four replications. Data (n = 40) were from the four field experiments conducted in three growing seasons in two locations in 2014.

For all parameters related to leaf N concentration, the narrow side generally had higher values than the wide side (Figs 4–6). The differences in Nw between the two sides of flag leaf were statistically significant only in the middle growing season in Wuhan (Table 3). For the -2^nd^ and -3^rd^ leaves, the two sides had statistically significant differences in Nw except for the early growing season. On average, Nw of the narrow side was 8.0%, 10.5%, and 11.4% higher than that of the wide side for the flag, -2^nd^, and -3^rd^ leaves, respectively. The difference in Na between the two sides was statistically significant only in the late growing season for the three leaf positions and in the middle growing season in Wuxue for -2^nd^ and -3^rd^ leaves. On average, Na of the narrow side was 4.7%, 9.5%, and 12.1% higher than that of the wide side for the flag, -2^nd^, and -3^rd^ leaves, respectively. The narrow side had statistically and significantly higher SPAD than the wide side regardless of growing seasons and locations. On average, SPAD of the narrow side were 2.3, 3.3, and 4.1 units higher than that of the wide side for the flag, -2^nd^, and -3^rd^ leaves, respectively.

**Table 3:** Leaf nitrogen concentration based on dry weight (Nw) and leaf area (Na), and chlorophyll meter reading (SPAD) of the wide and narrow sides of leaf blade in the top three leaves at heading stage.

Location	Season	Flag		-2^nd^		-3^rd^		Average	
		Wide	Narrow	Wide	Narrow	Wide	Narrow	Wide	Narrow
					Nw (%)				
Wuxue	Early	4.03 a	4.15 a	4.04 a	4.21 a	3.62 b	3.84 a	3.90 b	4.07 a
Wuxue	Middle	3.18 a	3.43 a	2.87 b	3.26 a	2.62 b	3.05 a	2.89 b	3.25 a
Wuhan	Middle	3.48 b	3.77 a	3.25 b	3.52 a	3.02 b	3.31 a	3.25 b	3.53 a
Wuxue	Late	3.77 a	4.16 a	3.52 b	3.97 a	3.26 b	3.61 a	3.52 b	3.91 a
	Mean	3.49 B	3.77 A	3.25 B	3.59 A	2.99 B	3.33 A	3.24 B	3.56 A
					Na (g m^-2^)				
Wuxue	Early	1.80 a	1.76 a	1.68 a	1.71 a	1.41 a	1.48 a	1.63 a	1.65 a
Wuxue	Middle	1.72 a	1.83 a	1.33 b	1.50 a	1.11 b	1.30 a	1.38 b	1.54 a
Wuhan	Middle	1.73 a	1.77 a	1.40 a	1.47 a	1.20 a	1.28 a	1.44 b	1.51 a
Wuxue	Late	1.59 b	1.71 a	1.26 b	1.46 a	1.07 b	1.24 a	1.31 b	1.47 a
	Mean	1.70 B	1.78 A	1.37 B	1.50 A	1.16 B	1.30 A	1.41 B	1.52 A
					SPAD				
Wuxue	Early	43.6 b	46.0 a	44.6 b	47.2 a	43.8 b	46.8 a	44.0 b	46.7 a
Wuxue	Middle	45.1 b	47.4 a	41.4 b	45.6 a	38.0 b	43.3 a	41.5 b	45.4 a
Wuhan	Middle	43.7 b	45.5 a	42.2 b	44.5 a	40.7 b	43.7 a	42.2 b	44.6 a
Wuxue	Late	40.3 b	43.6 a	39.1 b	43.0 a	36.9 b	41.3 a	38.7 b	42.6 a
	Mean	43.5 B	45.8 A	41.6 B	44.9 A	39.3 B	43.4 A	41.5 B	44.7 A

The experiments were conducted in the early, middle, and late growing seasons in Wuxue County and Wuhan City, Hubei Province, China in 2014.

Within a row for each leaf position, means followed by different letters are significantly different according to the least significant difference (LSD) at the 0.05 probability level.

Overall, the differences in the four N-related parameters between the two sides of leaf blade were the largest in -3^rd^ leaf followed by -2^nd^ and flag leaves (Table 3). As leaf width, thickness, and SLW, flag leaf had the highest Nw, Na, and SPAD followed by -2^nd^ and -3^rd^ leaves.

## Discussion

Leaf lateral asymmetry in width was reported previously in rice [14,15], however, the information on the magnitude of differences between the two sides of leaf blade is not available in the literature. Here, we report that the leaf width of the wide side was about 17% higher than that of the narrow side across large number of rice varieties grown under different conditions. We also observed that the wide or narrow side was located on the right or left side alternately depending on leaf position. Therefore, we describe leaf lateral asymmetry with wide and narrow sides instead of right and left sides in this paper.

Leaf lateral asymmetry in leaf thickness was firstly reported in rice by Chen et al. [20] who found 27.6 to 46.0% differences in leaf thickness between the two sides of leaf blade in two varieties. In our study, the wide side had on average 24.5% higher leaf thickness than the narrow side. Furthermore, leaf thickness measured by Chen et al. [20] was higher than the values of this study because different instruments were used to measure leaf thickness in the two studies. A special modified instrument that was used by Chen et al. [20] was an instrument for measuring the thickness of steel objects (John Bull, England). This instrument has a contact area of 0.5 cm^2^ between the leaf surface and the probe. Therefore, large veins could not be avoided during the measurement of leaf thickness by Chen et al. [20]. The instrument used in this study was developed by China Jiliang University [7]. This instrument has a very small contact area between the leaf surface and the probe, and the tip of the probe was spherical so that large veins can be avoided during the measurement of leaf thickness.

Because the instrument for measuring leaf thickness is not readily available, many crop researchers use SLW as an indication for leaf thickness [9,10]. Correlation coefficients (*r* ^2^) between SLW and leaf thickness ranged from 0.43 to 0.67 in rice leaf [7]. In this study, there was very small difference in SLW between the wide and narrow sides of leaf blade especially for -2^nd^ and -3^rd^ leaves despite of large difference in leaf thickness between the two sides.

Leaf lateral asymmetry was studied only on the top three leaves at heading stage in this study because the top three leaves of rice plants have greater contribution to yield formation than other leaves [3,23]. Among the top three leaves, flag leaf had the highest values in all measured morphological and physiological traits. However, the magnitude of leaf lateral asymmetry in -3^rd^ leaf was the greatest followed by -2^nd^ and flag leaves especially for N-related parameters. The cause for different magnitudes in leaf lateral asymmetry across leaf positions is not clear and it is beyond the scope of this study. Leaf lateral asymmetry in leaf width, leaf thickness, Nw, and SPAD was also observed on leaves at tillering and panicle initiation stages (7^th^ to 11^th^ leaves counting from the bottom of plants) in separate experiments.

SPAD provides a simple, quick, and nondestructive method for estimating leaf N status [13]. Therefore, it is used to guide the timing and amount of N topdressing in rice crop [12]. In addition, SPAD is commonly used for facilitating research in the areas of plant nutrition, crop physiology and plant eco-physiology [12,24]. SPAD readings are usually taken on one side of midrib around the midpoint of leaf length [13]. In this study, we found that the narrow side had SPAD values on average 3.2 units higher than the wide side. This suggests that SPAD reading should be taken entirely on either wide or narrow side, preferably on wide side in order to minimize the interference of midrib on SPAD measurement.

On average, the narrow side had 9.9%, 7.8%, and 7.7% higher Nw, Na, and SPAD than the wide side, respectively. To our knowledge, this is the first report on leaf lateral asymmetry in leaf N status. Close correlations between leaf photosynthetic rate and leaf N concentration have been reported in many species [25]. All data of Nw, Na, and SPAD indicated that the narrow side of leaf blade had higher leaf N and chlorophyll concentration than the wide side, suggesting higher leaf photosynthetic rate in the narrow side. Leaf density is defined as the ratio of dry weight to volume and can be calculated by dividing SLW by leaf thickness [26]. Lateral symmetry in SLW and asymmetry in leaf thickness suggested an asymmetry in leaf density with a higher density in the narrow side than in the wide side. The higher leaf density in the narrow side can be caused either by more closely packed mesophyll cells [27] or by aggregation of substances such as nonstructural carbohydrates (NSC) and minerals [28]. Closely packed mesophyll cells will result in a lower intercellular air space fraction of leaf volume and a lower chloroplast surface area fraction of mesophyll surface area [29], which will potentially restrain CO_2_ diffusion inside leaves [30] and subsequently restrict photosynthesis. The difference in leaf anatomy especially mesophyll cell arrangement and the size of intercellular spaces between the two lateral halves in rice leaves needs further investigation. NSC consists 28% [31] or even more than 40% [28] of total leaf dry mass. NSC aggregation in mesophyll cells may cause mechanical damage in chloroplasts and inhibit photosynthesis [32]. Therefore, it is necessary to confirm leaf lateral asymmetry in photosynthesis using the method of chlorophyll fluorescence image in the future studies. Genetic control of leaf lateral asymmetry in width was studied in rice by Obara et al. [15]. Environmental effects on leaf lateral asymmetry in width were studied in other species [16–18]. The study of leaf lateral asymmetry in photosynthesis may have implication to the improvement of rice productivity through crop breeding and crop management strategies.

In summary, leaf lateral asymmetry was observed in leaf width, leaf thickness, Nw, Na, and SPAD while leaf lateral symmetry was observed in SLW in rice. The wide side had higher leaf thickness than the narrow side whereas the narrow side had higher Nw, Na, and SPAD than the wide side. The magnitude of leaf lateral asymmetry for leaf width, leaf thickness, Nw, Na, and SPAD was the largest in -3^rd^ leaf followed by -2^nd^ and flag leaves.
